# The use of PEEK in digital prosthodontics: A narrative review

**DOI:** 10.1186/s12903-020-01202-7

**Published:** 2020-08-02

**Authors:** Ioannis Papathanasiou, Phophi Kamposiora, George Papavasiliou, Marco Ferrari

**Affiliations:** 1 Department of Prosthodontics, Dental School, National and Kapodistrian University of Athens, Athens, Greece; 2 Department of Prosthodontics and Dental Materials, School of Dental Medicine, University of Siena, Siena, Italy

**Keywords:** Digital prosthodontics, Computer-assisted-design/ computer-assisted-manufacturing, Polyetheretherketone, Dental prostheses, Clinical applications

## Abstract

**Background:**

Advanced computer-aided design and computer-aided manufacturing (CAD-CAM) technology led to the introduction of an increasing number of machinable materials suitable for dental prostheses. One of these materials is polyetheretherketone (PEEK), a high performance polymer recently used in dentistry with favorable physical, mechanical and chemical properties. The purpose of this study was to review the current published literature on the use of PEEK for the fabrication of dental prostheses with CAD-CAM techniques.

**Methods:**

Electronic database searches were performed using the terms “PEEK”, “CAD-CAM”, “dental”, “dentistry” to identify studies related to the use of PEEK for the fabrication of CAD-CAM prostheses. The search period spanned from January 1990 through February 2020. Both in vivo and in vitro studies in English were eligible. Review articles and the references of the included publications were searched to identify relevant articles.

**Results:**

A great number of in vitro studies are available in the current literature pointing out the noticeable properties of PEEK. The use of PEEK has been recommended for a wide range of CAD-CAM fabricated fixed and removable dental prostheses. PEEK was additionally recommended for occlusal splints, intra-radicular posts, implant abutments, customized healing abutments and provisional restorations. However, only a few clinical studies were identified.

**Conclusions:**

PEEK could be considered as a viable alternative for CAD-CAM fixed and removable dental prostheses to well-established dental materials. Due to the scarcity of clinical data, clinical trials are needed to assess the long-term performance of PEEK prostheses.

## Background

The rapid evolution of computer-aided design and computer-aided manufacturing (CAD-CAM) technology led to the introduction of new materials that could be precisely milled for the fabrication of dental prostheses [1]. Polyetheretherketone (PEEK) is a linear, aromatic, semi-crystalline thermoplastic, high performance polymer recently used in dentistry as a framework material for metal-free fixed dental prostheses [1, 2], removable dental prostheses [3], implant-supported fixed prostheses [4], implant-retained overdentures [5], endocrowns [6] and resin bonded fixed dental prostheses [7]. It has been also used for the manufacturing of dental implants [8], implant abutments [9], healing abutments [10] and occlusal splints [11]. PEEK is a material with high biocompatibility, good mechanical properties, high temperature resistance, chemical stability, polishability, good wear resistance, low plaque affinity and high bond strength with veneering composites and luting cements. Compared to rigid framework materials such as zirconium oxide and metal alloys, PEEK has a low modulus of elasticity of 4 GPa, and is as elastic as bone, providing a cushioning effect and reduction of stresses transferred to the abutment teeth [12–20]. Although PEEK is becoming widespread in clinical practice, only a few studies are available focusing on the use of this material for CAD-CAM prostheses. The purpose of this study was to review the current published literature on CAD-CAM PEEK dental prostheses.

## Methods

A literature search was conducted using several electronic databases (MEDLINE, PubMed, Scopus) for studies related to the use of PEEK for the fabrication of CAD-CAM prostheses. The search terms that the reviewers used alone or in combination were “PEEK”, “CAD-CAM”, “dental”, “dentistry”. The search period spanned from January 1990 through February 2020. Initially, the reviewers screened titles, abstracts or both for relevance according to the inclusion criterion, which was studies on PEEK prostheses. Both in vitro and in vivo studies were eligible in this review. Studies in a language other than English or without an English-language abstract were excluded. The reviewers obtained the full text of all relevant articles that passed the first review phase. The option of related-articles searches was also used. At this point, reviewers searched the references of the selected studies and review articles to identify relevant articles, which provided with few papers from years before 1990 as well. Reviewers tabulated data from the included studies. The following information was obtained from the included publications: author(s), year of publication, study design, type of PEEK prosthesis or application, number of specimens or patients and main outcomes/findings.

## Results

### Fixed dental prostheses (FDPs)

PEEK is a relatively new material with favorable mechanical properties and good bonding with veneering composite materials that fulfills the basic requirements to be used as a framework material for fixed dental prostheses [20–22]. Table 1 and Table 2 summarize the main findings of the in vitro and in vivo studies included in this review.

**Table 1:** In vitro studies included in the review

Study (year)	Application	Materials tested	Outcomes
Stawarczyk et al. (2015) [20]	FDPs	CAD-CAM milled PEEK Pressed pellet PEEK Pressed granular PEEK (*n* = 15/group)	Higher mean fracture load (2.354 N) for milled FPDs than those pressed from granular PEEK (1.738 N)
Stawarczyk et al. (2013) [22]		CAD-CAM PEEK (*n* = 225)	Μean fracture load of 1383 N Plastic deformation starting approximately at 1200 N
Niem et al.(2019) [23]		CAD-CAM PEEK Zirconia Lithium disilicate glass-ceramic (*n* = 10/group)	PEEK exhibited higher modulus of resilience than lithium disilicate Comparable to that of gold alloy
Niem et al. (2019) [24]		CAD-CAM PEEK Ceramic Composite and Polymer-based materials (n = 10 /group)	Flexural strength and modulus of elasticity of PEEK not significantly influenced by thermocycling
Liebermann et al. (2016) [25]		PEEK Hybrid material Composite resins PMMA-based materials (*n* = 40/group)	PEEK demonstrated: The lowest solubility and water absorption Similar hardness parameters to PMMA-based materials
Taufall et al. (2016) [26]		CAD-CAM PEEK veneered with different methods (digital veneering, conventional veneering with crea.lign, conventional with crea.lign paste, and pre-manufactured veneers) (*n* = 30/group)	The digital veneering showed the highest fracture load resistance
Cekic-Nagas et al. (2018) [27]		CAD-CAM PEEK PMMA Composite resin and fiber-reinforced composite materials (*n* = 7/group)	Highest load bearing capacity for PEEK
Wimmer et al. (2016) [28]		CAD-CAM PEEK Nanohybrid composite PMMA-based material (*n* = 12/ group)	Significantly higher wear resistance for PEEK
Wachtel et al. (2019) [29]	IFDPs	CAD-CAM PEEK screw-retained crowns on titanium implants (n = 10)	Favorable fracture mode for PEEK compared to conventional materials Coronal displacement of bending points No screw loosening or veneer fracture
Sirandoni et al. (2019) [30]		CAD-CAM PEEK PMMA Zzirconia Co-Cr Ti	Highest deformation for PEEK and PMMA frameworks that decreased von Mises stresses in the frameworks, implants and abutments PEEK exhibited critical tensile stress values in the trabecular bone
Nazari et al. (2016) [31]		CAD-CAM PEEK Zirconia Nickel-chromium alloy (n = 10/group) 3-unit IFDPs on two implants	Failure loads: Zirconia 2086 ± 362 N nickel-chromium alloy 5591 ± 1200 N PEEK 1430 ± 262 N
Elsayed et al. (2019) [32]		CAD-CAM PEEK Zirconia Lithium disilicate crowns supported by titanium and zirconia implant abutments (*n* = 8/group)	High fracture resistance of PEEK crowns, comparable to zirconia and lithium disilicate
Jin et al. (2019) [33]		CAD-CAM PEEK and titanium frameworks veneered with composite resin *n* = 20/group	PEEK exhibited Higher shear bond strength than Ti, good marginal fit and fracture resistance (1518 N)
Preis et al. (2017) [1]		CAD-CAM PEEK Zirconia-reinforced lithium silicate ceramics Composite resins Zirconia (n = 8/group)	PEEK molar implant-supported crowns showed lower fracture resistance than zirconia crowns Total failure rate of PEEK screw-retained frameworks veneered with composite paste
Yilmaz et al. (2018) [34]		Seven different CAD-CAM HPPs 100% PEEK 80% PEEK with 20% filler 80% PEKK with 20% filler Ceramic reinforced PEEK Interlaced fiberglass and resin Fiber-composite material New generation cubic zirconia 3Y-TZP Zirconia	Higher fracture resistance for zirconia implant-supported frameworks with cantilevers than PEEK-based materials
Ghodsi et al. (2018) [35]		CAD-CAM PEEK Zirconia Composite (n = 12/group)	No clinically acceptable marginal gaps for PEEK No significant differences observed in retention forces
Zeighami et al. (2019) [36]		CAD-CAM PEEK Zirconia, Composite (n = 12/group)	Better marginal adaptation for zirconia than PEEK
Chen et al. (2019) [37]	RPDs	CAD-CAM PEEK Co-Cr Ti alloys	PEEK caused lower stresses on periodontal ligament and higher stresses on the mucosa
Tribst et al. (2020) [38]		PEEK Polyamide Polyoxymethylene Gold alloy Titanium CoCr	Polyoxymethylene and PEEK exhibited the lowest retentive forces
Peng et al. (2019) [39]		PEEK CoCr alloy	No significant difference in the long-term deformation
Muhsin et al. (2018) [40]		CAD-CAM PEEK granular PEEK Co-Cr casting alloy (n = 10/group)	Higher retentive force for milled PEEK clasps than thermopressed clasps Higher retentive forces for PEEK clasps at deeper undercuts with a thicker clasp design than Co-Cr clasps after 3 years of fatigue simulation
Negm et al. (2019) [41]		CAD-CAM Milled PEEK Thermo-pressed PEEK (n = 10/group)	Higher fit and trueness for directly milled frameworks
Arnold et al. (2018) [42]		CAD-CAM Milled PEEK Cast metal frameworks with different techniques (n = 12/group)	PEEK RPD frameworks have better precision and fit than metal frameworks fabricated using different techniques
Hada et al. (2020) [43]	Complete denture framework	PEEK Fiber-reinforced composite Nano-zirconia cobalt-chromium-molybdenum alloy (*n* = 6group)	PEEK provides lower reinforcement than the other materials
Emera et al. (2019) [5]	Double-crown-retained Removable Dental Prostheses	Zirconia or PEEK primary crowns Zirconia or PEEK secondary crowns	Telescopic attachments fabricated from zirconia primary crowns and PEEK secondary crowns exhibited the lowest stresses transmitted to the implants
Schubert et al. (2019) [44]		Implant-supported zirconia primary crowns with electroformed secondary crowns or CAD-CAM PEEK secondary crowns (n = 10/group)	Stable retentive force values over 10 years of simulated aging for PEEK secondary crowns
Merk et al. (2016) [45]		Zirconia primary crowns Secondary PEEK crowns of different taper and manufacturing methods; milled from PEEK blanks; thermo-pressed from PEEK pellets; thermo-pressed from granular PEEK (n = 10/group)	Fabrication method and taper angle had no consistent effect on retentive forces within different groups
Stock et al. (2016) [46]		Zirconia primary crowns Secondary PEEK crowns of different taper and manufacturing methods; milled from PEEK blanks; thermo-pressed from PEEK pellets; thermo-pressed from granular PEEK (n = 30/group)	Milled 0° tapered PEEK crowns presented the lowest retention force Milled 2° tapered PEEK crowns had the highest retention force values Retention force of pressed PEEK not influenced by the taper angle Decrease of retention after the first twenty pull-off cyclew for pressed PEEK
Wagner et al. (2018) [47]		PEEK telescopic crowns and cobalt chrome copings of different taper and manufacturing methods (n = 10/group)	Stable retention load values for each test group
Stock et al. (2016) [48]		Milled PEEK primary and cobalt-chromium (CoCr), zirconia (ZrO2) and galvanic (GAL) secondary crowns with three different tapers (n = 30, 10/taper)	Milled PEEK can be used as primary crown material with high retentive forces in combination with secondary zirconia, cobalt-chromium or electroformed crowns
Benli et al. (2020) [11]	Occlusal splint	CAD-CAM PEEK Vinyl acetate Polymethyl methacrylate Polycarbonate Polyethyleneterephthalate (n = 12/group)	After chewing simulation PEEK occlusal splints exhibited lower loss of volume and lower roughness alteration compared to other CAD-CAM materials
Benli et al. (2020) [49]	Intra-radicular posts	Milled PEEK Glass-fiber Cast-metal (n = 20/group)	PEEK posts exhibited the highest tensile bond strength and the lowest surface roughness
Kaleli et al. (2018) [9]	Implant abutments	PEEK and zirconia customized abutments	Finite element analysis showed higher stress values in restorative crowns for PEEK abutments
Abdullah et al. (2016) [50]	Provisional crowns	PEEK VITA CAD Temp Telio CAD-Temp Protemp 4	PEEK demonstrated superior fit and fracture strength than other materials

**Table 2:** Clinical studies included in the review

Study (Year)	Study design	Intervention	Outcome
Parmigiani-Izquierdo et al. (2017) [51]	Case report	Zirconia implants restored with milled PEEK frameworks veneered with composite resin for the replacement of upper molars	Cushioning of occlusal loads while chewing Viable solution for patients with intolerance to metal alloys
Cabello-Dominguez et al. (2019) [52]	Case report	Monolithic zirconia fixed prosthesis in the maxilla and PEEK framework with gingival composite resin combined with lithium disilicate crowns in the mandible for the rehabilitation of a completely edentulous patient	The reduced weight and modulus of elasticity of PEEK could reduce the risk of mechanical complications Higher cost than metal-ceramic or metal-acrylic restoration
Zoidis (2018) [4]	Case report	PEEK implant framework material in combination with prefabricated PMMA veneers for the fabrication of a complete maxillary arch implant-supported fixed restoration	Esthetic outcome comparable with that of ceramic restoration After 2 years, no sign of screw loosening, veneering material chipping, wear, or staining
Harb et al. (2019) [53]	Case report	Milled PEEK framework combined with acrylic resin denture teeth and heat-cured acrylic resin denture for Kennedy Class I RPD fabrication	Adequate fit and good patient satisfaction in terms of function and esthetics
Costa-Palau et al. (2014) [54]	Case report	Milled PEEK framework for the fabrication of a maxillary obturator prosthesis	Compared to conventional obturators PEEK frameworks permit the fabrication of lighter prostheses with improved retention, function and esthetics
Mangano et al. (2019) [55]	Clinical study	Combining Intraoral and Face Scans for the Design and Fabrication of CAD-CAM PEEK Implant-Supported Bars for Maxillary Overdentures (15 patients)	After a year in function 100% implant survival 80% success rate
Spies et al. (2018) [56]	Case report	Implant-supported overdenture with the receptor part of the bar milled from PEEK polymerized into a zirconia framework for the rehabitation of an edentulous patient	High patient satisfaction with function and esthetics after 6 months
Hahnel et al. (2018) [57]	Case report	Primary CoCr copings and secondary CAD-CAM PEEK framework veneered with composite resin for the fabrication of double-crown-retained interim removable dental prosthesis	Biocompatibilityand low weight no complications after 3 months
Siewert (2018) [58]	Case report	Primary zirconia copings and secondary PEEK framework veneered with monolithic zirconia for the rehabilitation of an edentulous patient with intolerance to titanium	High chewing comfort Dampening of chewing forces
Beretta et al. (2019) [10]	Randomized clinical trial	Comparison of CAD-CAM fabricated customized healing abutments and standard healing caps placed at the surgical stage for the creation of the desired emergence profile (n = 10/group)	After a healing period of 1–3 months Patients with PEEK customized healing abutments showed higher functional implant prosthodontics score (FIPS) and lower numerical rating scale (NRS) values

Although there are no clinical reports on CAD-CAM fabricated PEEK FDPs available in the literature, Stawarczyk et al. in an in vitro study found that three-unit fixed dental prostheses milled using CAD-CAM technology from pre-pressed PEEK blanks showed lower deformation and higher fracture loads (2354 N) than those pressed in granular form (1738 N) [20]. Furthermore, several in vitro studies claimed that PEEK could be a viable alternative for single crowns and fixed dental prostheses [22–26]. Three-unit PEEK frameworks demonstrated deformation of the FDPs at 1200 N and fracture in the connector of the FDPs at 1383 N [22]. If 870 N is considered the average maximum posterior mastication force [59] PEEK could be regarded as a suitable material for restorations in load bearing areas. Moreover, the fracture resistance of the CAD-CAM milled PEEK FDPs is much higher than those of lithium disilicate glass-ceramic (950 N), alumina (851 N) and zirconia (981-1331 N) [60, 61]. Another in vitro study testing different types of inlay-retained FDPs, found that PEEK had the highest load bearing capacity compared to PMMA, composite resin paste and fiber-reinforced composite materials [27].

Compared to zirconia, lithium disilicate, and a high-content gold alloy, PEEK presented a higher value for the modulus of resilience than lithium disilicate, comparable to that of gold alloy, indicating a high capability to elastically absorb destructive fracture energy [23]. Based on the stress-strain curves observed, a high capacity to dissipate energy plastically was also found. Furthermore, PEEK has a low modulus of elasticity (4 GPa) compared to chrome-cobalt alloys (220 GPa), gold alloys (91 GPa), zirconia (220 GPa), alumina (314 GPa) and lithium dissilicate (95 GPa), zirconia reinforced lithium silicate (70 GPa), Feldspathic porcelain (48,7 GPa) [62]. A 3D-Finite Element Analysis of monolithic full posterior crowns revealed that materials with higher elastic modulus present higher tensile stress concentration on the crown intaglio surface and higher shear stress on the cement layer that could facilitate crown debonding in oral conditions [62]. Due to its low modulus of elasticity, PEEK allows the absorption of functional stresses by deformation and acts as stress breaker reducing forces transferred to the abutment teeth [2, 6]. For this reason, two clinical reports suggested the use of a pressed PEEK based framework veneered with light polymerized composite resin for the fabrication of single crown or endocrown in cases of weakened or severely damaged abutment tooth, patient’s allergy to metals and parafunctional habits [2, 6].

In an attempt to assess longevity of restorative materials, Niem et al. evaluated the influence of thermocycling on mechanical properties of ceramic, composite and polymer-based materials. Flexural strength and modulus of elasticity of PEEK were not significantly influenced by thermocycling, indicating the material’s ability to preserve its properties [24]. After aging in different solutions, PEEK demonstrated the lowest solubility and water absorption values compared to composite resins, a hybrid material and PMMA-based materials and similar hardness parameters to PMMA-based materials [25].

PEEK frameworks have a grayish-brown or pearl-white opaque color and need to be veneered with a composite resin. Several studies on bond strength of PEEK with composite resins have proposed different pre-treatments such as airborne-particle abrasion, silica coating [63], piranha-etching [64], sulfuric acid [65], phosphoric acid or argon plasma [66] with conflicting results. However, most of the studies concluded that reliable bond strength to composite veneering resins and luting cements can be achieved when PEEK surfaces are pretreated and conditioned using adhesive systems containing methylmethac-monomers, such as Signum PEEK bond and Visio.link [67–71].

Several veneering methods are available; CAD-CAM fabricated veneers, conventional veneering with light polymerized composite resin and pre-manufactured veneers. Taufall et al. evaluated the fracture load of PEEK three-unit fixed dental prostheses veneered with different methods. The highest fracture load values were found for digital veneers, indicating that digital veneering is more reliable than conventional techniques. Adhesive failures were most common for pre-manufactured veneers while cracks in the pontic region starting from the connector area were observed for digital veneers and conventional composite resin [26].

An in vitro study evaluated the colorimetric properties of different veneering materials on PEEK, zirconium oxide (ZrO_2)_, cobalt–chromium–molybdenum alloy (CoCrMo), and titanium oxide. PEEK showed comparable results when compared to well established core materials such as ZrO_2_ and CoCrMo with respect to the CieLab-System parameters of the assemblies and the modification of the CieLab-System parameters for each veneering material [72].

Additional advantages of PEEK are the low abrasiveness to enamel and the high wear resistance. In an in vitro study, Wimmer et al. found significantly higher wear resistance for PEEK than a nanohybrid composite and a poly (methyl methacrylate) material when loaded laterally and comparable wear of enamel antagonists [28].

The overall conclusion of the previous studies is that PEEK can be used for CAD-CAM FDPs due to its good mechanical and bonding properties, although clinical evidence needs to be improved.

### Implant-supported fixed dental prostheses (IFDPs)

Frameworks for implant-supported fixed dental prostheses are typically fabricated by casting metal alloys or milling either titanium or zirconia. However, two recent clinical reports have presented PEEK frameworks veneered with composite resin as a solution for IFDPs, suitable for patients experiencing metal allergies [4, 51]. Another clinical report suggested the use of monolithic zirconia fixed prosthesis in the maxilla and PEEK framework with gingival composite resin combined with lithium disilicate crowns in the mandible for the rehabilitation of a completely edentulous patient. PEEK frameworks have reduced weight and higher elasticity than zirconia frameworks, which could reduce the risk of mechanical complications, but this solution has a higher cost compared to conventional metal-ceramic or metal-acrylic restorations [52].

Due to its low elastic modulus PEEK provides a cushioning effect on occlusal forces. When such an elastic framework is combined with materials with low elastic modulus such as poly (methyl methacrylate) (PMMA) pre-fabricated veneers or veneering composite resin, it will further reduce occlusal forces to the restoration and the opposing dentition. Therefore, the use of PEEK could be advantageous for IFDPs where proprioception is reduced by the absence of periodontal ligaments and eliminate mechanical complications such as veneer fractures and clicking sound during function reported for metal-ceramic or monolithic zirconia restorations [4, 52]. In agreement with this statement, an in vitro study evaluating screw-retained PEEK crowns on titanium implants found favorable fracture mode for PEEK compared to conventional materials while bending points were displaced coronally, providing protection from damage to the implant and the abutment screws [29]. No screw loosening, or veneer complication was found. On the other hand, the use of rigid frameworks fabricated by metal or zirconia could lead to plastic deformation of the implant shoulder [73, 74]. These results are in consistency with the in vitro study of Kaleli et al. reporting higher stress values of zirconia customized abutments on implant components, crown and cortical bone, compared to PEEK customized abutments [9]. Moreover, a three-dimensional finite element analysis on different framework materials for implant-supported fixed mandibular prostheses found the highest deformation for PEEK and PMMA frameworks that decreased von Mises stresses in the frameworks, implants and abutments. However, PEEK frameworks showed critical tensile stress values in the trabecular bone, while ZrO_2_, Co-Cr, and Ti reached stress values in the bone within physiologic limits [30].

Adequate fracture resistance is required to ensure good long-term outcomes of implant-supported prostheses. An in vitro study evaluating three-unit IFDPs on two implants fabricated by zirconia, nickel-chromium alloy, or PEEK found failure loads of 2086 ± 362 N, 5591 ± 1200 N and 1430 ± 262 N, respectively [31]. However, the fracture strength reported for PEEK prostheses was higher than the physiological maximum posterior masticatory of 870 N [59]. Thus, PEEK prostheses have been considered capable to withstand occlusal forces in the molar region while the failure mode observed was adhesive between veneering composite and framework [31]. El Sayed et al. found high fracture resistance of PEEK crowns, comparable to zirconia and lithium disilicate crowns supported by titanium and zirconia implant abutments [32]. Adequate fracture resistance values of 1518 ± 134 N have also been found by Jin et al. in an in vitro study [33]. On the other hand, Preis et al. in a fatigue testing of PEEK molar crowns either bonded or screw-retained found lower fracture resistance than zirconia ones, while a total failure rate was observed for PEEK frameworks veneered with composite paste used for screw-retained restorations, indicating that the insertion of screw channels weakened the PEEK frameworks [1]. Moreover, zirconia implant-supported frameworks with cantilevers showed higher fracture resistance than PEEK-based materials [34].

Clinically acceptable marginal gap is considered to be less than 120 μm while acceptable marginal adaptation has been suggested to be between 50 and 100 μm. Ghodsi et al. found in an vitro study no clinically acceptable marginal gaps for PEEK and composite implant-supported copings while zirconia had the best marginal and internal adaptation. However, no significant differences were observed in retention forces among materials evaluated with pull-out test [35]. In another study the marginal adaptation of PEEK implant-supported frameworks before and after cementation has been on the borderline of clinical acceptability but with significantly higher marginal discrepancy than zirconia frameworks [36]. On the other hand, Jin et al. found good marginal fit values of 19 ± 4 μm for PEEK three-unit implant-supported frameworks [33], in agreement with the results of Wachtel et al. who reported no bacterial leakage of screw-retained PEEK crowns during masticatory simulation [29].

Chipping of the veneering materials is a common complication of IFDPs with a titanium framework. A previous in vitro study reported stronger bonding of PEEK three-unit implant-supported frameworks (31.1 ± 3.5 MPa) with composite resins than titanium frameworks (20.5 ± 1.8 MPa), concluding that PEEK could be used as an alternative framework material to titanium [1]. Durable bonding of PEEK with composite resins permits, also, easy intraoral repair of PEEK restorations with composite resin in case of chipping [4].

Additional advantages of PEEK is its radiolucency, which may facilitate cement removal and screw loosening diagnosis and its low specific weight permitting the construction of lighter prostheses [4]. Due to the white color of PEEK frameworks, the grayish appearance of metal frameworks can be eliminated and a high esthetic outcome can be achieved in combination with composite veneering materials. Furthermore, PEEK has good biocompatibility combined with low water solubility and high chemical and thermal stability. Thus, PEEK prostheses could be suitable for patients experiencing allergies to metals and metallic taste and for patients demanding metal-free restorations [4]. However, more clinical studies are needed to evaluate the behavior of these new material.

### Removable dental prostheses (RDPs)

Computer-aided design and computer-aided manufacturing (CAD-CAM) techniques can be also used to fabricate RDP frameworks. A previous clinical report has suggested PEEK frameworks combined with acrylic resin denture teeth and heat-cured acrylic resin denture bases as an alternative to conventional Co-Cr frameworks [53]. PEEK presents favorable properties such as excellent biocompatility, good mechanical properties, good thermal and chemical resistance, white color and low specific weight that permit the fabrication of lighter metal-free RPDs eliminating the esthetically unacceptable display of metal claps and the risk for metallic taste and allergies of conventional RDP metal frameworks [3, 53]. Another study described the use of milled PEEK frameworks for the fabrication of a removable maxillary obturator prosthesis [54]. Both studies reported high patient satisfaction with regard to esthetics, retention and comfort [53, 54].

Due to its high elasticity, PEEK could reduce stresses and distal torque on the abutment teeth during function [3]. In agreement with this statement, a three-dimensional finite element analysis of Chen et al. found that PEEK frameworks caused lower stress values on periodontal ligament than cobalt-chromium and Ti-6Al-4 V alloy. Thus, PEEK RPDs could be recommended for patients with poor periodontal conditions [37]. However in the same study, it was found that PEEK caused the highest stresses on the mucosa and the greatest displacement on the free-end that could lead to pain, advanced bone resorption, denture base failure and compromised chewing efficiency [37]. The authors concluded that PEEK should be used with caution in distal extension RDPs. Moreover, compared to metal frameworks, PEEK ones showed significantly lower internal stresses.

Retention force and fatigue resistance are crucial factors for RDP clasps. Two in vitro studies found that PEEK clasps exhibited lower retentive force than Co-Cr alloy clasps [38]. However, retention force values of PEEK clasps were considered sufficient for clinical use, while Tannous et al. recommended the use of 0,5 mm undercuts [75]. No significant differences were found in deformation of PEEK and metal clasps after fatigue testing [39]. On the other side, Tribst et al. claimed that PEEK should not be used for clasp fabrication because stress values during removal of clasps with higher undercuts are higher than the material strength [38]. With respect to fabrication method of PEEK frameworks, milled PEEK clasps demonstrated higher retentive force than thermo-pressed ones. Both milled and thermo-pressed PEEK clasps showed higher retaining forces at deeper undercuts with a thicker clasp desing than Co-Cr clasps after 3 years of fatigue simulation [40].

CAD-CAM PEEK RDP frameworks can be fabricated by several methods such as direct milling of PEEK blanks or 3D printing of a resin/wax pattern framework which is then thermo-pressed using the conventional lost-wax/resin technique [41]. Clinically acceptable fit values were found for both techniques but directly milled PEEK frameworks had higher fit and trueness values than indirectly fabricated frameworks. In agreement with this result, Arnold et al. found that directly milled PEEK RPD frameworks have better precision and fit (43 ± 23 mm horizontal, and 38 ± 21 mm vertical) than cast metal frameworks fabricated using the conventional lost-wax casting technique, indirect rapid prototyping or direct rapid prototyping. This was attributed to the high-quality finish achieved by the milling technique [42].

PEEK could also be used as a framework material for complete dentures in order to decrease denture deformation responsible for midline fractures [43, 76]. However, PEEK frameworks with a thickness of 1 mm could offer only a slight reinforcement to complete dentures, while more rigid materials such as fiber-reinforced composite (FRC), nano-zirconia (N–Zr), cobalt-chromium-molybdenum alloy provide greater reinforcement with a thickness of 0,5 mm [43]. This finding can be explained by the similar deformation of PEEK and PMMA due to their compararable elastic moduli which are 4 GPa [19] and 2.7 GPa [77], respectively. Muhsin et al. evaluated denture bases fabricated by milled or thermo-pressed PEEK and PMMA. The results of this in vitro study showed that PEEK denture bases had higher impact and tensile strength than PMMA. Thus, PEEK could be regarded as a material suitable for denture bases providing resistance to notch concentration and fracture [78]. Futhermore, two in vitro studies found better stain resistance and lower surface roughness after polishing of PEEK materials compared with PMMA [79, 80].

Furthermore, a few studies stated that PEEK may be used as an attachment retaining implant-supported overdentures [55, 56]. In a clinical study of Mangano et al., 15 fully edentulous patients were rehabilitated with a maxillary overdenture supported by 4 implants and CAD-CAM fabricated PEEK bar. After a year in function, no implants were lost and an 80% success rate for implant-supported overdentures was found [55]. A clinical report also suggested the use of an implant-supported overdenture with the receptor part of the bar milled from PEEK polymerized into a zirconia framework for the rehabitation of an edentulous patient. The authors reported high patient satisfaction with function and esthetics after 6 months [56].

### Double-crown-retained removable dental prostheses

A case report of Hahnel et al. suggested the use of primary metal copings and secondary CAD-CAM PEEK framework veneered with composite resin for the fabrication of double-crown-retained interim removable dental prosthesis [57]. Another case report described the use of primary zirconia copings and secondary PEEK framework veneered with monolithic zirconia for the rehabilitation of an edentulous patient with intolerance to titanium [58]. The study reported high chewing comfort and patient satisfaction with low weight, very good fit and retention. According to Emera et al. telescopic attachments fabricated from zirconia primary crowns and PEEK secondary crowns could also be a viable solution for retaining implant overdentures, providing a reduction of stresses transmitted to the implants due to the stress-breaking capacity of PEEK [5].

Several in vitro studies tested retention forces of double crown systems with primary zirconia crowns and secondary PEEK crowns. An in vitro study found that secondary PEEK crowns provide stable retentive forces after 10 years of simulated aging and comparable values at baseline with well-established electroformed crowns [44]. Another advantage of digitally fabricated telescopic crowns is that in case of loss of retention or other technical complication any part of the double crown system can be reproduced using the stored data. Merk et al. evaluated retention between zirconia primary crowns and secondary PEEK crowns of different taper and manufacturing methods; milled from PEEK blanks; thermo-pressed from PEEK pellets; thermo-pressed from granular PEEK. The outcomes of the study showed that the fabrication method and taper angle had no consistent effect on retentive forces within different groups. However, with regard to retention, PEEK could be considered as viable solution for double-crown-retained RDPs with primary zirconia crowns [45]. In a similar study, Stock et al. found that milled 0° tapered PEEK crowns presented the lowest retention force, whereas milled 2° tapered PEEK crowns had the highest retention force values. The retention force of the pressed PEEK crowns was not influenced by the taper angle [46]. However, pressed PEEK groups showed a decrease of retention after the first twenty pull-off cycles. The explanation given by the authors was that the higher elasticity of pressed PEEK leads to a slight deformation during the removal of the secondary crowns. Thus, precise milling of PEEK blanks could be a more predicable technique for double crown systems [46]. The same conclusions were reached by Wagner et al. who studied the retention between PEEK telescopic crowns and cobalt chrome copings of different taper and manufacturing methods [47]. Another in vitro study demonstrated that milled PEEK could be also used as primary crown material with high retentive forces in combination with secondary zirconia, cobalt-chromium or electroformed crowns [48].

### Occlusal splints, intra-radicular posts, implant abutments, healing abutments and provisional restorations

The use of PEEK was additionally recommended for CAD-CAM fabricated occlusal splints. An in vitro study of Benli et al. found lower loss of volume and change in roughness for PEEK occlusal splints after chewing simulation compared to other CAD-CAM materials such as vinyl acetate (EVA), polymethyl methacrylate (PMMA), polycarbonate (PC), and polyethyleneterephthalate (PETG) [11]. It was also claimed that milled PEEK intra-radicular posts could be an alternative to glass-fiber and cast-metal posts. According to an in vitro study, PEEK posts presented higher tensile bond strength than metal and glass-fiber posts when used with the appropriate surface treatment and adhesive system [49]. Previous studies evaluated the performance of PEEK for CAD-CAM fabricated implant abutments, customized healing abutments and provisional crowns [9, 10, 50]. A finite element analysis comparing PEEK and zirconia customized abutments found higher stress values in restorative crowns for PEEK abutments [9]. A randomized clinical trial of Beretta et al. evaluated the use of CAD-CAM fabricated customized healing abutments and standard healing caps placed at the surgical stage for the creation of the desired emergence profile. After a healing period of 1–3 months PEEK customized healing abutments created a natural gingival architecture and required less prosthetic steps for the formation of the emergence profile compared to the use of standard healing caps [10]. Last but not least, Abdullah et al. in an in vitro study compared CAD-CAM provisional crowns with direct provisional crowns. The materials used were VITA CAD Temp, PEEK, Telio CAD-Temp, and Protemp 4. Based on the results of this study, digitally produced PEEK provisional restorations demonstrated better fit and fracture strength than conventional provisional crowns [50].

## Conclusions

Several in vitro studies and clinical reports suggested that PEEK could be suitable for CAD-CAM fabricated fixed and removable dental prostheses due to its favorable mechanical, chemical and physical properties. However, further in vitro and clinical studies are needed to evaluate the long-term performance of these prostheses before PEEK can be safely recommended as an alternative to well-established prosthodontic materials.

## Data Availability

Not applicable.

## Abbreviations

PEEK: Polyetheretherketone
CAD: Computer-assisted-design
CAM: Computer-assisted-manufacturing
FDP: Fixed Dental Prosthesis
IFDP: Implant-supported Fixed Dental Prosthesis
RDP: Removable Dental Prosthesis
PMMA: Poly (methyl methacrylate)
Ti: Titanium
ZrO2: Zirconia
CoCr: Cobalt-Chromium
CoCrMo: Cobalt-Chromium-Molybdenum
